# Gene Therapy for Wet Age-Related Macular Degeneration

**DOI:** 10.3390/bioengineering12101072

**Published:** 2025-10-02

**Authors:** Normila Barthelemy, Jayanth Sridhar, Jesse D. Sengillo

**Affiliations:** 1 University of Miami Leonard L. Miller School of Medicine, Miami, FL 33125, USA; 2 Bascom Palmer Eye Institute, Miami, FL 33136, USA; 3 Olive View, UCLA Medical Center, Los Angeles, CA 91342, USA

**Keywords:** wet AMD, anti-VEGF, gene therapy, pharmacokinetics, adeno-associated viral (AAV) vectors, CRISPR-cas9

## Abstract

The prevalence of wet age-related macular degeneration (AMD) in the US is expected to increase to 82 million by 2050. Addressing the specialized needs for this population will become increasingly challenging as prevalence rises. Frequent anti-vascular endothelial growth factor (anti-VEGF) injections have been the recourse for this population; however, the burden wet AMD places on patients underscores the critical need for durable therapeutic approaches. Gene therapy is a bioengineered treatment that has transformed the management of previously untreatable disorders. Ongoing advancements and refinements in its biomechanism could lead to more sustainable treatment options for wet AMD. In this article, we provide recent updates on gene therapy trials for wet AMD.

## 1. Introduction

Age-related macular degeneration (AMD) is a progressive and debilitating condition. Its multifaceted pathophysiology is influenced by both genetic predisposition and environmental factors [1,2]. The natural progression of AMD leads to two distinct advanced clinical presentations. One is dry age-related macular degeneration, where vision loss is characterized by progressive retinal atrophy. The other form is neovascular age-related macular degeneration (nAMD or often referred to as wet AMD), which is identified by exudative choroidal neovascularization. When nAMD is left untreated, it can lead to rapid vision loss. It is well known that environmental factors such as smoking and obesity influence the progression of AMD [1], in addition to genetic mutations that confer increased risk [3]. The products of the Complement Factor H (*CFH*) gene have also been implicated in inflammatory and immune pathways, which further support the role of inflammatory indicators such as the tumor necrosis factor (TNF)-α, complement components and interleukin-1 and 6 (IL-1, IL-6) in the pathogenesis of nAMD [4].

The formation of choroidal neovascularization depends on the release of vascular endothelial growth factor (VEGF) from the retinal pigment epithelium toward the inner choroid, where high levels of VEGF receptors are located [5]. In classic ischemic retinal diseases, VEGF is induced by hypoxia. However, in nAMD, local immune reactivity and inflammatory markers are the predominant triggers. VEGF interacts with the endothelial cell receptors VEGFR receptor 1 and VEGFR receptor 2, which triggers an intracellular signal transduction cascade [6].

The introduction of anti-vascular endothelial growth factor (anti-VEGF) agents has been revolutionary in the field of retinal medicine, particularly for neovascular retinal diseases. For patients affected with wet AMD, the variable frequency and cost of anti-VEGF injections driven by treatment regimens are important economic considerations. Additionally, the economic impact of wet AMD is significant, with anti-VEGF accounting for over $40 billion annually and 12% of the US Medicare part B budget [7,8]. Holistic cost-saving analysis studies across longer-acting anti-VEGF agents found that they differ in acquisition cost and in the frequency of required injections [9]. When considering projected injection frequency, wholesale acquisition cost, time, and distance travelled, the triannual visit cost for ranibizumab was $72,080, $39,946 for aflibercept and $33,265 for farcimab.

Gene therapy is an innovative approach that has been applied to retinal disease, demonstrating promising efficacy in untreatable retinal diseases and other ocular conditions [10]. The first transformative step in ophthalmic gene therapy was the use of a recombinant adeno-associated virus (AAV) carrying *RPE65*-complementary DNA (cDNA) for patients with RPE65-associated Leber’s congenital amaurosis (LCA) [11]. This subretinal approach laid the foundation for the field. Since then, the sustained clinical improvements and favorable immunological profile of this therapy have inspired advances in vector design and driven clinical trials for Stargardt disease, choroideremia, retinitis pigmentosa, and Usher syndrome [10,12,13,14,15].

Over the past few years, significant progress has been made in innovative treatments for wet AMD that aim to provide long-lasting effects compared with frequent anti-VEGF injections. Gene therapy for wet AMD has shown particularly promising results. Based on cohort size, efficacy, and stage of advancement, Ixo-vec (ADVM-022), ABBV-RGX-314, and 4D-150 have been the most promising candidates (Table 1). Many of these clinical trials have completed phase 2 testing with phase 3 outcomes forthcoming. The following article reviews the current state of the art for gene therapies applied to AMD.

## 2. Gene and Cell Therapy

The major gene therapy modalities currently investigated in clinical trials are gene replacement, gene editing, ribonucleic acid (RNA) modulation, cell-based therapy, and oncolytic virotherapy [16]. The mechanism of gene replacement uses a viral vector to deliver a functional copy of a defective gene to restore normal protein function. Similarly, oncolytic virotherapy uses viral vectors to selectively lyse cancer cells. Gene editing and RNA modulation techniques directly modify the genetic material to alter its expression. 

The current gene therapies in clinical trials for wet AMD mainly employ gene replacement or RNA modulation to suppress retinal vascular endothelial growth factor (VEGF). The main vectors used in these trials are adeno-associated viral (AAV) vectors, which offer several advantages. AAV vectors minimize immune reactivity and can target specific cell populations via engineered serotypes [17]. For many inherited ocular conditions being approached with gene therapy, the etiology is predominantly monogenic [11,18], which makes AAV vectors more appealing given their small but precise genetic payload capacity. In addition, their episomal DNA is stable without integrating into the host genome, theoretically decreasing oncogenicity [19]. This allows them to persist in postmitotic cells such as retinal cells, but progressively lost in dividing cells [20,21].

Gene replacement and gene editing are common techniques employed in the therapies for monogenic IRDs. Monogenic inherited retinal diseases (IRDs) are characterized by a single defective gene, which presents a more defined roadmap for therapeutic development. The mendelian inheritance pattern makes it easier to target the root cause and delay disease progression. Bioengineering challenges are possibly less demanding for IRD gene therapy. Selecting a broad promoter and a vector capable of delivering a single gene payload is often sufficient to achieve pan-retinal expression in many conditions. 

In contrast, nAMD, requires complex vector engineering that also avoids excessive VEGF inhibition, which can lead to retinal atrophy. Unlike monogenic IRDs, gene therapy for AMD does not aim to correct a specific genetic mutation. Instead, it provides sustained delivery of therapeutic proteins that can alter the disease course.

**Figure 1:**
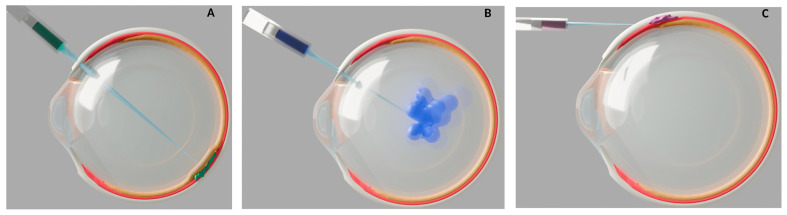
Schematic representations of ocular injection techniques for nAMD gene therapy. Cross-section illustrations of the human eye show three methods of delivering bioengineered gene therapy for neovascular age-related macular degeneration (nAMD), with relevant anatomical layers color coded: retina (orange), choroid (red), sclera (white). (**A**) Subretinal injection: The therapeutic agent (green) is delivered via a subretinal approach, using a syringe. (**B**) Intravitreal injection: The therapeutic agent (blue) is injected into the vitreous cavity through an intravitreal approach. (**C**) Suprachoroidal injection: The therapeutic agent (purple) is delivered into the suprachoroidal space.

Another layer of complexity for nAMD gene therapy is the delivery method. Subretinal injection can reduce the immune response risk due to the blood–retina barrier, which normally safeguards the retina from the systemic immune system [22] (Figure 1A). However, in nAMD this barrier may be compromised, and the surgical procedure itself confers risks of infection, hemorrhage, and retinal detachment to name a few.

A less invasive delivery approach is intravitreal injection, which can be performed in outpatient settings. However, this may increase the chance of exposure to systemic circulating antibodies [23] (Figure 1B). From a bioengineering aspect, intravitreal delivery also reduces vector efficacy, due to the inner limiting membrane limiting the penetration of vectors to outer retinal layers [24].

Suprachoroidal injections may offer a balanced option by reducing the immune response risks compared to an intravitreal delivery and potentially allowing outpatient administration (Figure 1C). However, this approach may have less pharmacokinetic advantage, as increased drug clearance through choroidal circulation can decrease the duration of treatment in suprachoroidal space [25]. This rapid clearance increases the risk that the vectors do not have sufficient time to transduce target tissues and exert their effects.

## 3. Phase 3 Clinical Trials

The clinical translation of AAV technology has facilitated a spectrum of trials for the treatment of wet AMD. The leading phase 3 gene therapy trials have been RGX-314, Ixo-vec(ADVM-022) and 4D-150.

RGX-314 is an AAV vector with serotype 8 (AAV8), containing cDNA encoding a monoclonal antibody fragment antigen binding (Fab) protein that mimics ranibizumab, and use a chicken β-actin promoter (CB7) [26]. Serotype 8 provides efficient transduction in murine retinal cells [27], has moderate neutralizing seroprevalence, and superior efficiency when crossing blood vessel barriers compared to other serotypes [28]. The CB7 promoter is effective for sustained gene expression [29]. The expressed Fab fragment serves as an antagonist, neutralizing VEGF-A. While ranibizumab targets all VEGF-A isoforms [30], no biophysical data are available on RGX-314’s anti-VEGF Fab fragment affinity, isoform specificity, or kinetic parameters. 

The phase 1/2a dose-escalation (NCT03066258) study of RGX-314 showed sustained RGX-314 protein concentrations in aqueous humor at the two year endpoint [26]. RGX-314 is delivered by a small-gauge vitrectomy, followed by a single subretinal injection without a pre-bleb. In this phase 1/2a dose escalation study of 42 participants with wet AMD, 13 participants experienced 20 serious adverse events (macula pigment mottling, macula-sparing retinal detachment, endophthalmitis, reduced visual acuity, recurrent transitional cell carcinoma, and the remaining were systemic events). In cohort three (6 × 10^10^ genome copies (GC)/eye) and four (1.6 × 10^11^ GC/eye), 50% gained ≥15 letters, respectively, at week 106.

RGX-314 phase 2 trials (AAVIATE and ATMOSPHERE) are evaluating a less invasive, office-based suprachoroidal delivery, without vitrectomy. According to Regenexbio, in AAVIATE (n = 116), there was a 60–80% decrease in the number of supplemental intravitreal anti-VEGF injections needed per year across dose cohorts. In cohorts receiving 6 × 10^10^ GC/eye, the mean BCVA was maintained at ±5 ETDRS letters or improved over two years, with the highest dose (1 × 10^12^ GC/eye) showing dose dependent efficacy [26]. However, multiple moderate treatment-emergent adverse events were reported, such as episcleritis and an increase in IOP. The ongoing phase 3 trials (ATMOSPHERE and ASCENT) with approximately 765 patients globally are evaluating suprachoroidal and subretinal RGX-314, respectively (NCT05407636).

Ixo-vec (ADVM-022) is an AAV vector with serotype 2/7m8 peptide (AAV2.7m8) that contains a CD11-optimized cDNA that encodes a complete aflibercept protein with its associated binding domain VEGF-A/B, placental growth factor and the fragment crystallizable region (Fc) portion of human immunoglobulin (IgG1) [31]. The 7m8 peptide insertion disrupts AAV2 binding to heparan sulfate proteoglycans in the inner limiting membrane (ILM) at the vitreoretinal junction [24]. It is believed that this disruption facilitates infusion into the retinal cells, based on its high transduction of retinal tissues [32,33]. It also helps reduce antibody recognition, given AAV2’s low neutralizing seroprevalence [28]. 

Results from the phase 1 and phase 2 studies (NCT03748784; NCT05536973) revealed that aflibercept levels in aqueous humor were stable and maintained through study completion. The intervention for the Ixo-vec (ADVM-022) trials includes administration of one intravitreal injection dosage in an outpatient setting with oral or topical steroids.

In the phase 1 dose-ranging (6 × 10^11^ vector genome(vg)/eye or 2 × 10^11^ vg/eye) study of 30 patients with wet AMD, 20% of patients in the higher dosage (6 × 10^11^ vg/eye) cohort and 7% in the lower dosage cohort experienced serious adverse events (cataract, uveitis, retinal detachment, or dry AMD progression). In the phase 2 randomized, double-masked, dose-ranging study of 60 patients with enhanced topical steroids, no serious adverse events were observed. There were two discontinuations due to unrelated adverse events (lung malignancy and cardiopulmonary arrest), and no systemic immunological reactions were observed in either study phases. 

In the phase 1 study, 80% of patients in the higher dose cohort were injection-free at the two years landmark and 53% of the lower dose cohort remained injection-free at two years. In both cohorts there was ≥80% reduction in annualized anti-VEGF injections. In the phase 2 study, 76% of patients in the 6 × 10^10^ vg/eye cohort remained injection-free at 26 weeks, whereas 83% of those in the 2 × 10^11^ vg/eye cohort remained injection-free at 26 weeks [34]. The current phase 3 (ARTEMIS) non-inferiority study evaluating the 6 × 10^11^ vg/eye dosage vs. aflibercept every eight weeks with prophylactic steroids is actively enrolling participants (NCT06856577) [35].

4D-150 is different from the other AAV vectors, because it is a synthetic AAV capsid (R100) and has a dual transgene payload. Similarly to Ixo-vec, it contains a codon-optimized cDNA encoding aflibercept, but it also encodes a miRNA-30 backbone targeting VEGF-C mRNA. With this additional feature it is able to antagonize four VEGF family members (A, B, C and PIGF) [36,37]. 

Although R100 is a relatively new engineered AAV variant, it has shown marked transduction efficiency in preclinical studies. In vitro functional assessment in human retinal cells, derived from induced pluripotent stem cells, revealed that R100 had a 2.6- to 3.75-fold higher transduction compared to AAV2 [38]. However, this assay lacks the complexity of human retinal tissue to effectively assess the biological challenges faced by vectors injected intravitreally, such as the ILM, vitreous humor, extracellular matrix, and human immunological response. In Cynomolgus macaques and African green monkeys, bilateral R100 resulted in widespread and stable transduction across all retinal cells, leading to clinical trials in human subjects [38]. 

The protocols for the 4D-150 clinical trials included the administration of a single intravitreal injection of 4D-150. In the phase 1 open-label dose, exploration (3 × 10^10^, 1 × 10^10^, and 6 × 10^9^ vg/eye) study of 15 patients with topical steroid taper post injection, no serious adverse events were observed. The 3 × 10^10^ vg/eye dosage was the most efficient with a mean central subfield thickness (CST) reduced by 92 µm at 36 weeks, and an 84% reduction in annualized anti-VEGF injections [39]. However, the results were worse for the 1 × 10^10^ dose cohort with a CST +38 ±37 µm at 24 weeks and stable for the 6 × 10^9^ vg/eye dose cohort with +3 ±25 µm [40]. 

In the randomized, controlled phase 2 (PRISM; NCT05197270) clinical trial, patients assigned to two treatment arms (1 × 10^10^ vg/eye and 3 × 10^10^ vg/eye) demonstrated satisfactory steroid compliance and no serious adverse events [41]. The current phase 3 (4FRONT-1; NCT07064759) trial will cover a more global population with over 800 patients, with a fixed 3 × 10^10^ vg/eye dosage as the intervention [42].

## 4. Phase 2 Clinical Trials

In addition to the phase 3 clinical trials discussed, several therapies are currently being investigated in the early stages of clinical development. LX102 and rAAV.sFlt-1 are currently in phase 2 clinical trials. 

LX102 uses an AAV vector with serotype 2, similar to Ixo-vec, with the gene coding for VEGF-Trap (aflibercept). This allows the specific inhibition of VEGF- A and PIGF, but not VEGF- C or D [43]. The intervention for the LX102 clinical trials includes a single intravitreal injection of aflibercept (2 mg/0.05 mL), followed by subretinal injection of LX102 dosages (2 × 10^10^ or 1.25 × 10^11^ vg) two weeks later. Although no LX102-related adverse events were reported, more than half of the nine patients in the study experienced procedure-related adverse events, such as conjunctival hyperemia, post-operative visual acuity reduction, increased intraocular pressure (IOP), and mild cell debris in the anterior vitreous [44]. Similarly to phase 1, the current phase 2 randomized, parallel-group (NCT06196840) trial of LX102 is actively recruiting local participants in China.

rAAV.sFlt-1 is composed of a recombinant adeno-associated virus serotype 2 (rAAV2) vector that contains the sFLT-1 transgene variant. The expression of this gene antagonizes VEGF-A, VEGF-B, and PlGF [45,46]. Preclinical studies of this new engineered vector have revealed sustained retinal preservation in transgenic mice [47]. 

In the phase 1 randomized controlled trial (NCT01494805) the injection involved a 23-gauge three-port pars plana vitrectomy with core vitreous removal and posterior vitreous detachment induction, followed by a subretinal delivery of 100 μL therapy (1 × 10^10^ or 1 × 10^11^ vg) via a 41G cannula. Although no systemic adverse events attributed to rAAV.sFLT-1 were noted, one patient developed a systemic immune response to AAV2 antigens and three of the six patients in the treatment cohorts experienced some adverse events (mild cell debris, subconjunctival and subretinal hemorrhage) [48]. In the combined phase 1/2a trial (NCT01494805) with only the high dose, 33 serious adverse events occurred over three years. Three cancer (breast cancer, colon cancer and lung cancer) diagnoses occurred during the trial, although two patients had a history of malignancy prior to starting the clinical trial. There were two non-lethal cardiac events, one transient choroiditis, one chronic eye inflammation, and the remaining serious adverse events were attributed to age-related infirmities. There were no statistically significant differences between the rAAV.sFlt-1 and control groups in visual acuity, retinal thickness, or sFLT-1 protein at any time point during the trial [49]. Given this lack of meaningful efficacy or safety benefits, the development of rAAV.sFlt-1 for wet AMD has been discontinued.

## 5. Phase 1 Clinical Trials

Wet AMD gene therapy trials that have started phase 1 include but are not limited to NG101, KH658, FT-003, KH631, and AAV2-sFLT01.

Similarly to RGX-314, NG101 is a recombinant adeno-associated virus serotype 8 (rAAV8) vector, but it encodes aflibercept instead of a Fab fragment similar to ranibizumab. A preclinical evaluation of this engineered vector showed effective transduction in vitro via non-retinal cells (CHO-K1 and HEK293 cells) and in vivo (subretinal injection of NG101 1 × 10^6^ to 1 × 10^9^ vg/eye; intravitreal aflibercept injection as a comparator) in a mouse model [50]. This bioassay of NG101 proved to be effective, with sustained expression of aflibercept from the vector persisting one year post injection in mice, although no additional long-term data are available in this mouse model. 

The phase 1 dose escalation study clinical trial (NCT05984927) for NG101 received an FDA fast track designation in November 2024 to enroll US- and Canada-based patients. The result of the safety and effectiveness of this gene therapy is expected in 2025.

KH658 is a recombinant AAV vector of an unspecified serotype that contains the transgene for VEGF receptor. Although there have been no published peer-review preclinical studies on KH658, it has started its phase 1 trial (NCT06458595).

Similarly to KH658, there is paucity of published peer-reviewed data on the therapeutic payload or biomechanism of FT-003. However, according to the global clinical-stage biotechnology company that engineered FT-003, the preliminary outcome of the phase 1 clinical study (NCT05611424) conducted in China is showing promising safety results. This limited clinical study of approximately 15 patients revealed that a single intravitreal injection of FT-003 was well tolerated with no serious adverse effect [51]. As of the last update, no safety or efficacy results have been published on ClinicalTrials.gov.

KH631 is composed of a rAAV8 vector with the transgene for the human VEGFR1/VEGFR2-Fc fusion protein that antagonizes all VEGF-A/B and PlGF with great affinity (inhibitory concentration (IC50) value of 269.2 pM). A pharmacokinetics and biodistribution assessment in rhesus monkey via subretinal injections of KH631 at 1 × 10^11^ vg/eye revealed that the transgene expression was higher in retinal tissue compared to vitreous [52]. Given the promising outcome of KH631, the phase 1 dose escalation study (NCT05672121) is underway to establish its safety and efficacy in humans.

Although many of these gene therapy trials have shown great potential for the future of wet AMD, AAV2-sFLT01 remained at phase 1 (NCT01024998), with no subsequent phase 2 or 3 trials initiated. AAV2-sFLT01 is composed of an AAV2 vector with a transgene encoding domain 2 of sFlt1 linked to a human Fc domain. Like RGX-314, this therapy uses the chicken β-actin promoter to drive gene expression and enable production of the therapeutic protein. sFlt1 naturally binds VEGF-A, VEGF-B, and PlGF [53]. Studies on its molecular and functional properties have shown that it exhibits a high affinity for this protein and that it binds VEGF-A with high affinity [54]. 

Preclinical studies on a mouse oxygen-induced retinopathy model have demonstrated adequate transduction effectivity of AAV2-sFLT01 [55]. However, the phase 1 dose-escalation trial revealed that only five patients in the highest-dosage cohorts (had detectable sFLT01 in their aqueous humor. Given AAV2’s poor neutralizing seroprevalence, only patients with low anti-AAV2 serum antibody titers achieved therapeutic sFLT01 levels in this study. This could also explain why only 4 of the 19 patients in this study had a sustained reduction in CST at 52 weeks [56]. The unreliable transgene expression and inferior outcome compared to anti-VEGF injection has halted the development of this gene therapy.

## 6. Emerging Molecular Editing Therapy for AMD: CRSPR-CAS13

The CRISPR/Cas gene-editing system is widely considered as one of the most efficient and innovative bioengineered tools in the field of genomics [57]. Its revolutionary status was officially affirmed in 2020, when Doudna and Charpentier were awarded the Nobel Prize in Chemistry for its discovery [58]. In addition, CRISPR-based therapies have been approved for sustained remission in both beta-thalassemia and sickle cell disease [59,60]. Currently, the CRISPR/Cas9 mechanism has not seen the same level of application as the traditional AAV vectors in wet AMD gene therapy research. 

HG202 is the only CRISPR-based gene therapy that is currently in clinical trials for wet AMD. Compared to the previously described AAV-based anti-VEGF gene therapies, HG202 is composed of an undisclosed AAV capsid that contains an RNA editing enzyme, Cas13Y, a type VI CRISPR system. This class 2 type VI RNA endonuclease directly degrades VEGF-A mRNA, preventing its expression. This mechanism enables persistent suppression of VEGF-A and its isoforms without relying on continuous transgene expression. Its RNA-targeting capabilities and efficacy have been investigated in transgenic mice [61,62]. However, there are no studies in non-human primates or on long-term durability of Cas13Y currently. HG202’s first-in-human phase 1 trial in China was initiated, and more recently, its phase 1 dose-escalation study (NCT06623279) has started in the US.

## 7. Weaknesses of Ocular Gene Therapy

One of the main weaknesses of ocular gene therapy has been immunological responses. Many nAMD clinical trials have attempted to mitigate inflammatory reactions by using different injection techniques, enhancing dose-ranging strategies with topical or oral steroids, and by addressing neutralizing seroprevalence of viral vectors. However, significant challenges to both the effectiveness and safety of gene therapy in nAMD remain. Some patients still develop autoantibodies and secondary autoimmune retinopathy after injections. 

The current landscape of ongoing clinical trials for neovascular age-related macular degeneration (nAMD) suggests a persistent emphasis on therapies targeting the VEGF pathway. This may have limited potential benefit to patients with a documented history of inadequate responses to anti-VEGF agents. XMVA09 represents an innovative approach by encoding a bispecific antibody designed to simultaneously inhibit both VEGF-A and ANG-2, offering a rationale for broader efficacy, including in VEGF-refractory cases. Nevertheless, several critical challenges remain. The recent investigator-initiated clinical trial assessed XMVA09 in only six nAMD patients, all of whom had a history of multiple anti-VEGF therapy injections in the past, but none had a documented history of resistance to prior anti-VEGF treatments [63]. It is difficult to determine whether therapeutic effects in these subjects were due to VEGF-A inhibition, ANG-2 inhibition, or synergism between both pathways. Future trials would benefit from dedicated cohorts of non-responders and treatment-naive patients to determine response rates in these groups.

In addition, there is uncertainty regarding the long-term results and durability of treatment. For example, the inferiority of AAV2-sFLT01 compared to current anti-VEGF therapies led to discontinuation of its clinical trial. This underscores the critical need to improve both surgical and minimally invasive delivery methods for therapeutic agents. This optimization should focus on injection approaches, vector dosing protocols, and formulation strategies to achieve maximum transduction efficiency and safety.

Although, these vectors are engineered for stable transduction in postmitotic cells; however, the therapeutic benefit may decline over time. Another important point to consider is the accessibility and economic aspect of gene therapy. Established anti-VEGF treatments currently available for nAMD already account for a considerable portion of the Medicare budget. Limited patient access and stringent insurance approval processes are key challenges that may hinder the widespread implementation of gene therapy.

Although these innovative therapies are impactful and important, we must consider the dependability of engineered gene editing therapies. The pathway to FDA approval for bioengineered therapies follows high standards, with strict manufacturing protocols, robust regulatory planning, and comprehensive review of all results before advancing through clinical phases. This approach is not intended to hinder scientific innovation, but rather to safeguard the efficacy and safety of these groundbreaking treatments. There are proven examples demonstrating the durability and effectiveness of gene therapies in clinical medicine. With appropriate regulatory oversight and safety planning, these treatments have already transformed the clinical management of conditions such as RPE65-associated LCA, hemophilia A, sickle cell disease, and beta thalassemia [11,60,64,65]. With ongoing refinements in gene therapy mechanisms, a major breakthrough for wet AMD is within reach. One that promises durable treatment efficacy and lasting sight preservation for an expanding patient population.

## 8. Summary Statement

In the above article we review the current state of the art for gene therapies applied to wet-AMD. The leading phase 3 gene therapy trials are RGX-314, Ixo-vec (ADVM-022), and 4D-150. LX102 and rAAV.sFlt-1 are at an earlier stage but may hold promising results. There are multiple wet AMD gene therapy trials that have just started their phase 1 clinical trials, including KH658, FT-003, NG101, KH631, and AAV2-sFLT01. However, HG202 is the only CRISPR-based gene therapy that is currently in clinical trials for wet AMD.

## Figures and Tables

**Table 1:** Overview of Wet AMD gene therapy drugs. VA: visual acuity; CST: Central Subfield Thickness.

Drug Name	Mechanism	Vector	Clinical Phase	Study Design	Population Type	Sample Size	Intervention	Primary Endpoint	Key Results
RGX-314	Gene Replacement	Non-replicating adeno-associated virus serotype 8	Phase 3	Quadruple, Randomized, Parallel Assign ment	Multicenter (US, Canada, France, Germany, Hungary, Italy, Japan, Puerto Rico, Spain, UK)	660	Subretinal Injection Suprachoroidal Injection	Mean Change from baseline BCVA	-Decrease Injection rate -Sustained improvement in BCVA
4D-150	Gene Replacement + RNA modulation	Non-replicating adeno-associated virus	Phase 3	Quadruple Randomized, Parallel Assignment	Multicenter (US, Puerto Rico, and Canada)	400	Intravitreal Injection	Mean Change from baseline BCVA	-Safe and tolerated -up to 2.5 years of sustained BCVA and CST Reduction
rAAV.sFlt-1	Gene Replacement	Non-replicating adeno-associated virus	Phase 1/2a	Single, Randomized, Parallel Assignment	Single site (Australia)	40	Subretinal Injection	Tolerability and safety	-51 Ocular AEs -No fluid control
AAV2-sFLT01	Gene Replacement	Non-replicating adeno-associated virus serotype 2	Phase 1	Open-Label Non-Randomized Dose Escalation Parallel Assignment	Multicenter (US)	19	Intravitreal Injection	Maximum tolerated dose	-44 AEs -55% with significant fluid control and VA improvement
Ixo-vec (ADVM-022)	Gene Replacement	Non-replicating Adeno-associated virus—2.7m8	Phase 3	Double Randomized Parallel Assignment	Multicenter (US)	284	Intravitreal Injection	Mean Change from baseline BCVA	-Safe and tolerated -up to 3.5 years of sustained fluid control
HG202	RNA modulation	Non-replicating adeno- associated virus	Phase 1	Open-Label Sequential Assignment	N/A	15	Subretinal Injection	Adverse Events Incidence	N/A
KH631	Gene Replacement	Non-replicating adeno-associated virus serotype 8	Phase 1	Open-Label Single Group Assignment	Multicenter (US & China)	42	Subretinal Injection	Adverse Events Incidence	N/A
KH658	Gene Replacement	Non-replicating adeno-associated virus	Phase 1	Open-Label Non-Randomized Sequential Assignment	Multicenter (US & China)	44	Suprachoroidal Injection	Tolerability and safety	N/A
FT-003	Gene Replacement	Non-replicating Adeno-associated virus—2.7m8	Phase 1/2	Open-Label Non-Randomized Sequential Assignment	Single site (China)	78	Intraocular Injection	Adverse Events Incidence	N/A
LX102	Gene Replacement	Non-replicating adeno-associated virus serotype2	Phase 2	Open-Label Randomized parallel-assignment	Multicenter (China)	50	Subretinal Injection	Change in VA	N/A
NG101 AAV	Gene Replacement	Non-replicating adeno-associated virus serotype 8	Phase 1/2a	Open-Label Dose Escalation	Multicenter (US & Canada)	18	Subretinal Injection	Adverse Events Incidence	N/A

## Data Availability

Not applicable.
